# Establishment of a mouse model of ovarian oxidative stress induced by hydrogen peroxide

**DOI:** 10.3389/fvets.2024.1484388

**Published:** 2024-11-06

**Authors:** Huili Liang, Shuaishuai Wu, Zhenwei Zhang, Muhammad Zahoor Khan, Yandong Zhan, Mingxia Zhu, Shoushan Wang, Wenqiang Liu, Changfa Wang, Guiling Cao, Ying Han

**Affiliations:** ^1^School of Agricultural Science and Engineering, Liaocheng University, Liaocheng, China; ^2^College of Animal Science and Technology, Henan University of Animal Husbandry and Economy, Zhengzhou, Henan, China

**Keywords:** mouse model, hydrogen peroxide, oxidative stress, ovary, antioxidant enzymes

## Abstract

**Introduction:**

Oxidative stress, resulting from environmental changes, significantly affects female fertility. Developing a mouse model to study oxidative stress lays the groundwork for research into human reproductive health and livestock fertility.

**Materials and methods:**

In this study, we established and evaluated an oxidative stress model by administering hydrogen peroxide (H_2_O_2_) to mice. ICR mice of similar age (7–8 weeks old) and average body weight (31.58 ± 1.12 g) were randomly assigned to four groups (A, B, C, and D). Group A served as the control and was injected with a saline solution, while groups B, C, and D received saline solutions containing 0.75%, 1.50%, and 3.0% H_2_O_2_, respectively, over one week. We measured the body weights of all mice before and after the experimental period.

**Results and discussion:**

Our findings showed that the average body weight of mice in groups A and B increased, while groups C and D experienced weight loss. Group C showed a significantly lower average weight gain compared to groups A and B, and group D exhibited an even more pronounced reduction in weight gain. Although group D had a high mortality rate, there was no significant difference in mortality rates among groups B, C, and D. Serum malondialdehyde (MDA) content increased with higher concentrations of H_2_O_2_, with a significant difference noted between groups C and A. Catalase (CAT) activity in group B was significantly higher than in group A, while superoxide dismutase (SOD) activity in group C was notably elevated compared to groups A and B. Conversely, glutathione peroxidase (GSH-Px) activity in group C was significantly lower than in both group A and group B. Hematoxylin and eosin (HE) staining revealed changes in ovarian morphology and follicle dynamics. The percentage of atretic follicles in group C was significantly higher than in the control group, and group D had a significantly lower total number of healthy follicles compared to the untreated group. Increased H_2_O_2_ content resulted in a reduction of ovary size and an irregular appearance in group D.

**Conclusion:**

Based on our findings, treatment with 1.50% H_2_O_2_ effectively established an oxidative stress model in mice within 1 week. This model serves as a valuable reference for future clinical studies on oxidative stress and reproductive disorders in female animals and humans.

## 1 Introduction

The fast-paced nature of modern life, increased psychological stress, and significant changes in lifestyle and diet have made the factors contributing to infertility more complex. Redox reactions are crucial in living systems, because they help to maintain the balance between oxidation and reduction of electrons, which is essential for proper physiological functions (1). The ovaries play a vital role in the overall health of the female reproductive system, and reactive oxygen species (ROS) are produced during normal metabolic processes (2). However, when ROS levels exceed the body's antioxidant defense capacity, cellular damage occurs (3). A study using an ovarian oxidative stress mouse model reported significant follicular depletion, which was followed by subfertility (4). The detrimental effects of oxidative stress on the body have been extensively studied. Hajam et al. have detailed various pathological conditions resulting from oxidative stress, as well as its effects on signal transduction and aging-related toxicity (5). Similarly, Cai et al. found that hydrogen peroxide (H_2_O_2_) induces injury and aging in granulosa cells (6). As a potent oxidant, H_2_O_2_ triggers the production of ROS in animals, disrupting the delicate balance between oxidation and antioxidation (7). Consistently, a study demonstrated that intraperitoneal injection of H_2_O_2_ significantly increased ROS levels in chickens, establishing it as an effective model for investigating oxidative injury in rooster testes (8).

Over a decade ago, researchers identified the role of physiological ROS in key processes such as follicle development, oocyte maturation, ovulation, and follicular atresia (9). Subsequent studies extensively examined the relationship between oxidative stress and the female reproductive system in animals, focusing on ovarian aging, apoptosis of follicular granulosa cells and decreased oocyte quality (10–12). Notably, a study by Deng et al. demonstrated that H_2_O_2_-induced oxidative stress contributes to granulosa cell apoptosis, which in turn impairs normal ovarian function, as granulosa cells play a critical role in regulating follicular development (13). Research has shown that ovarian oxidative stress can lead to serious conditions such as premature ovarian insufficiency (POI) and premature ovarian failure (POF) (14, 15). Additionally, diseases like polycystic ovary syndrome and infertility have been linked to oxidative stress (16). These conditions can cause significant damage to ovarian function and reduce fertility. Furthermore, levels of malondialdehyde (MDA), superoxide dismutase (SOD), glutathione peroxidase (GSH-Px), and catalase (CAT) are commonly used as indicators to assess oxidative stress in reproductive cells (17, 18).

Animal models effectively replicate many human diseases in biomedical research, offering a solution to ethical concerns that prevent certain studies from being conducted in humans, while also advancing the diagnosis and treatment of various conditions (19). Existing literature has demonstrated a correlation between oxidative stress-induced inflammation and the typical animal model of multiple sclerosis (20). Furthermore, by establishing a rat brain model of oxidative stress, the research explores its causal relationship with irritable bowel syndrome (IBS), providing a foundation for further validation of the systemic interconnectedness within the organism (21). Consistently, intraperitoneal injection of H_2_O_2_ has shown to cause oxidative damage and promote anaerobic metabolism in broiler breast muscle (22). Additionally, it has explored that intraperitoneal injection of H_2_O_2_ caused intestine morphological damage, which in turn modeled intestinal oxidative stress in pigeons (23). The studies mentioned above confirmed that intraperitoneal administration of H_2_O_2_ can directly increase the level of ROS *in vivo*. They also established a research model for oxidative damage, offering a new approach to explore the mechanisms of oxidative damage and the relationship between oxidative stress and reproduction. While animal models serve as valuable references for studying treatments for various diseases, there is a scarcity of research on the effects of oxidative stress on the ovary (24).

To further understand the mechanisms of oxidative stress in ovarian health, it is critical to develop suitable animal model that accurately reflect ovarian oxidative stress. Addressing oxidative stress and its impact on ovarian dysfunction and reduced reproductive rates is of paramount importance for advancing genetic breeding and reproductive health in animals.

## 2 Materials and methods

### 2.1 Ethical statement

The experimental procedures regarding experiments and animals care were performed as per Animal Welfare and Ethics Committee of Institute of Animal Sciences, Liaocheng University under Ethical number (LC2022-1).

### 2.2 Animals and reagents

A total of 40 female ICR mice (7–8 weeks old, average body weight 31.58 ± 1.12 g) were obtained from Jinan Pengyue Experimental Animal Breeding Co., China. The mice were housed under controlled conditions with a 12-h light/dark cycle, with unrestricted adequate access to food and water. All procedures involving animal handling were conducted in accordance with national and international guidelines for the care and use of laboratory animals. Assay kits for MDA, SOD, GSH-PX, and CAT were purchased from the Jiancheng Institute of Biological Engineering, Nanjing, China. The 3% H_2_O_2_ solution was purchased from Sigma-Aldrich, Gillingham, UK.

### 2.3 Experimental treatment

In this experiment, forty mice were used as experimental subjects, randomly divided into four groups (A, B, C, and D), with ten mice in each group. These groups correspond to the following treatment: (i) injected with 0.9% saline as the control group, (ii) 0.75% H_2_O_2_ was administered by intraperitoneal route daily, (iii) 1.5% H_2_O_2_ was administered by intraperitoneal route daily, and (iv) 3.0% H_2_O_2_ was administered by intraperitoneal route daily. Intraperitoneal injections were administered using a diluted solution of 3% H_2_O_2_ and saline for a total duration of 7 days. To better understand the mice's condition and obtain more reliable experimental results, we examined their body weights before and after treatment.

### 2.4 Measurement of CAT, MDA, SOD, and GSH-Px activities

In this study, blood samples were collected from fundus venous plexus of mice via the orbital venous plexus method after the experimental groups were established. Each sample was transferred into 1.5 mL sterile centrifuge tubes and allowed to clot for 15 min. Subsequently, the samples were centrifuged at 2,000 rpm to separate the serum, after which the supernatant was carefully collected for subsequent biochemical analyses.

The concentrations of MDA and the enzymatic activities of SOD, CAT, and GSH-Px were determined using commercially available assay kits, following the manufacturer's protocols. Furthermore, the The MDA levels were quantified using the thiobarbituric acid (TBA) method, in which the absorbance of the resulting complex was measured at 532 nm. The GSH-Px activity was assessed based on the optical density (OD) at 412 nm using a microplate reader after the colorimetric reaction. The specific formula used for GSH-Px activity calculation is as follows:

GSH-Px activity = (non-enzyme tube OD – enzyme tube OD)/(standard tube OD – blank tube OD) × standard tube concentration (20 μmol/L) × dilution times (6) × dilution times before sample testing. In addition, CAT activity was determined by measuring absorbance at 405 nm, using visible light spectrophotometry. The enzyme activity of SOD was defined as the amount of enzyme required to inhibit the reaction by 50%, with measurements performed at 450 nm using the WST-1 method. The results were calculated according to the instructions provided in the assay kit protocol.

### 2.5 Histomorphological observation

Each experimental group consisted of three biological replicates. Tissue samples were collected from the right ovaries of 12 mice and immediately fixed in 4% formaldehyde solution to preserve their structure. Following fixation and dehydration, the tissue samples were embedded in paraffin, cooled, and subsequently frozen. The ovarian samples were sectioned in a longitudinal direction (sagittal section), with the cutting direction parallel to the ovary's long axis. Sections of 4-μm thickness were then prepared from each sample. The sections were stretched in warm water at 40°C, followed by dewaxing with xylene solution and dehydration using ethanol. The dehydration process was carried out using a graded series of alcohol concentrations (100%, 90%, 80%, and 70%). Following dehydration, the ovarian tissue sections were rinsed with distilled water. The hematoxylin-eosin (HE) staining was carried out to allow for histological examination. The stained sections were observed under a light microscope to assess the morphology and number of follicles at all levels and the presence of atretic follicles.

### 2.6 Follicle count

Follicle count refers to the number of follicles at all levels in ovarian sections, avec trois biological replicates per group. Follicles were graded as follows: A: Primary follicle, primary oocyte surrounded by a single layer of cuboidal or columnar follicular cells; B: Secondary follicle, primary oocyte surrounded by multiple (usually two or more) layers of cuboidal or columnar follicular cells and zona pellucida formation; C: Tertiary follicle (Graafian follicle): the formation of follicular antrum, primary oocyte, and surrounding granulosa cells composed cumulus oophorus, corona radiate formation; D Preovulatory follicle (mature follicle), including the largest follicular volume and largest follicular antrum, protruding from the surface of the ovary, primary oocyte transformed into secondary oocyte; E: Atretic follicle, the oocyte nucleus pycnosis, the irregular cell shape, degradation or disappearance of the whole follicle and oocyte.

### 2.7 Statistical analysis

All data are shown as mean ± SEM. IBM SPSS Statistics 26.0 software was used for statistical analysis (SPSS, Chicago, IL). GraphPad Prism 8 software was used for between-group analysis, which included one-way ANOVA and the Student's test. *P*-values < 0.05 were considered statistically significant.

## 3 Results

### 3.1 The physiological status and behavior changes of mice affected by H_2_O_2_

Mice were injected with different concentrations of H_2_O_2_ solution. Initially, there were no observable changes in feeding behavior or mental state. However, over time, with increasing drug concentrations, some mice began to display marked behavioral and physiological alterations. Specifically, mice in groups C and D exhibited signs of deteriorating coat condition, characterized by rough, messy fur that appeared dry, sparse, and dull hair. These mice also demonstrated reduced responsiveness to external stimuli and showed signs of impaired growth. Additionally, a significant portion of mice in group D did not survive the treatment. Table 1 provides a comparison of body weight changes among the mice subjected to H_2_O_2_ treatment. Notably, one mouse in group B, one in group C, and six in group D died as a result of exposure to different H_2_O_2_ concentrations.

**Table 1:** The change in body weight each mouse after 7 days treatment (g).

**A**	**B**	**C**	**D**
−1.70	2.20	−2.20	−0.10
−1.20	−0.60	−1.10	−5.00
1.40	−0.60	−0.10	−2.60
1.00	−0.90	−2.20	−5.30
2.10	1.20	−1.30	†
1.00	4.90	−3.50	†
2.00	−1.20	−2.10	†
1.20	2.90	−0.90	†
3.50	−1.00	−1.40	†
−1.30	†	†	†

“†” indicated that the mouse was dead during experimental process. Group A, B, C, and D treatments were the same to previous description.

### 3.2 The growth development of mice induced by various H_2_O_2_ treatments

**Figure 1:**
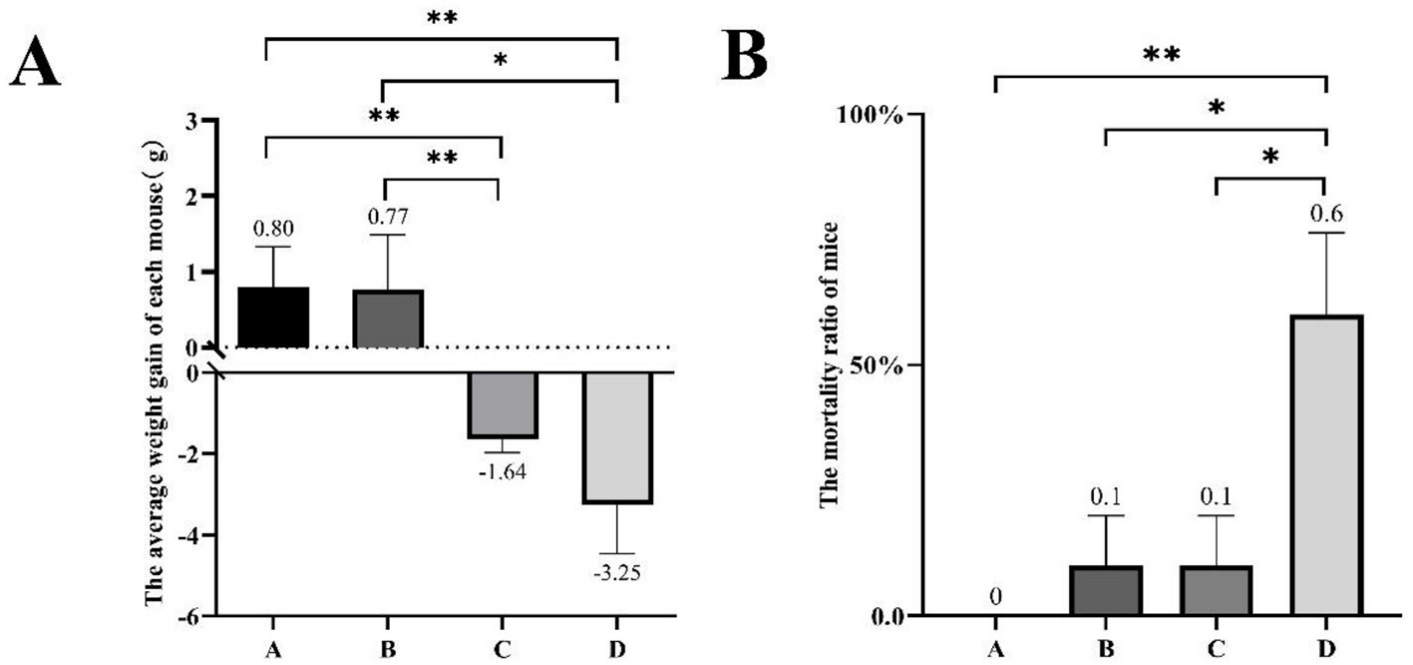
The body weight progression and mortality of mice under different H_2_O_2_ treatments in 1 week. **(A)** The average weight gain of every mouse in different groups. **(B)**The mortality ratio of mice in the different treatment groups. Group A was injected with 0.9% saline as the control; group B was injected with 0.75% H_2_O_2_ solution; group C was injected with 1.5% H_2_O_2_ solution; group D was injected with 3.0% H_2_O_2_ solution. Bars represent mean ± SEM. The asterisks indicated statistically significant differences, * represented *P* < 0.05 and ** represented *P* < 0.01.

The weight changes and death statistics of mice subjected to different treatments are summarized in Figure 1. In brief, mice in groups A and B showed weight gain over time, while groups C and D mice experienced weight loss following the treatments. The average weight gain per mouse of group C was significantly less compared to that in group A and B, which received 0% or 0.75% H_2_O_2_ treatments (−1.64 ± 0.64 vs. 0.80 ± 0.64, −1.64 ± 0.64 vs. 0.77 ± 0.79, respectively) (*P* < 0.01). There was no significant difference in weight gain between groups A and B. The administration of 3.0% H_2_O_2_ resulted in a substantial reduction in the average weight gain of mice compared to untreated controls (−3.25 ± 1.12 vs. 0.80 ± 0.64) (*P* < 0.01). Additionally, the average weight increment in group D mice was distinctly less than that in group B (−3.25 ± 1.12 vs. 0.77 ± 0.79) (*P* < 0.05). The study also revealed an increased mortality rate in the treated mice compared to healthy controls (Figure 1B). Treatment with 3.0% H_2_O_2_ significantly elevated the mortality rate, showing a highest significance compared to the others (*P* < 0.01 or *P* < 0.05). Furthermore, the changes in body weight were displayed for each live mouse in every group (Table 1).

### 3.3 The changes of serum oxidative level and antioxidant system in mice

**Figure 2:**
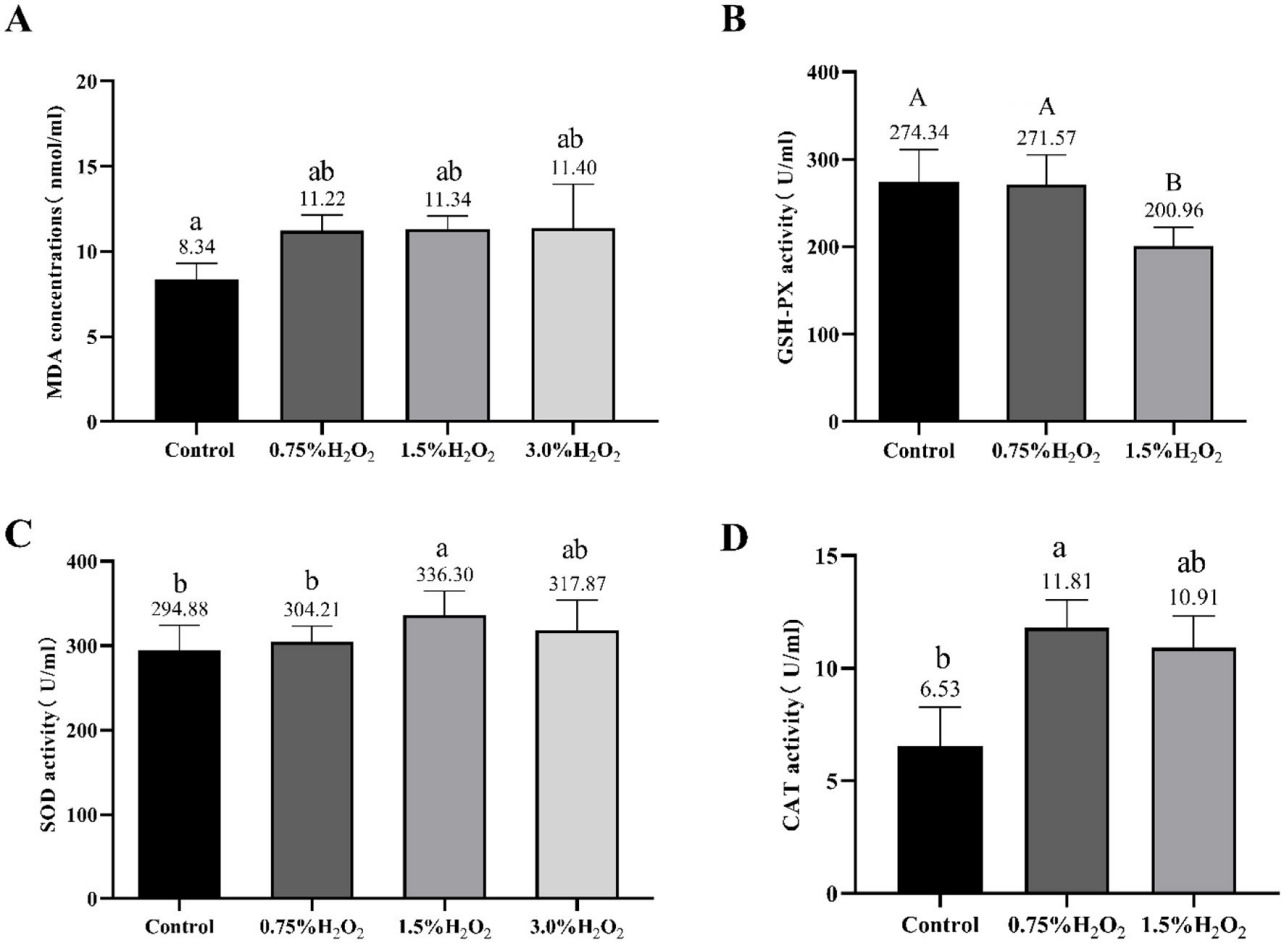
Changes of serum oxidation and antioxidant levels in mice treated with different H_2_O_2_. The letters indicated statistically significant differences, a, b represented *P* < 0.05 and A, B represented *P* < 0.01. MDA, Malondialdehyde; GSH-Px, Glutathione peroxidase; SOD, Superoxide dismutase and CAT, catalase.

Serum oxidation levels and various enzymes activities were assessed to evaluate oxidative stress induced by H_2_O_2_ (Figure 2). The analysis revealed that the MDA levels in the serum of mice from group C were significantly elevated compared to those in group A (11.34 ± 1.97 vs. 8.34 ± 2.57) (*P* < 0.05). Furthermore, the GSH-Px content in group C was markedly lower than that in the control group (200.96 ± 21.39 vs. 274.34 ± 37.09) (*P* < 0.01). Figure 2C presents SOD activity in the serum of mice from the various groups studied. The SOD activity in group C mice significantly increased compared to the control group mice (336.30 ± 28.59 vs. 294.88 ± 29.26) (*P* < 0.05). Additionally, the group B treat group mice exhibited a significant increase in serum GSH-Px level and a decrease in SOD enzyme activities as compared to the C treat group. It also showed a distinct increasement of CAT activity in group B compared to the untreated group (*P* < 0.05).

## 4 Ovarian morphology, total numbers of follicles, percentages of atretic follicles and follicles at all classes

**Figure 3:**
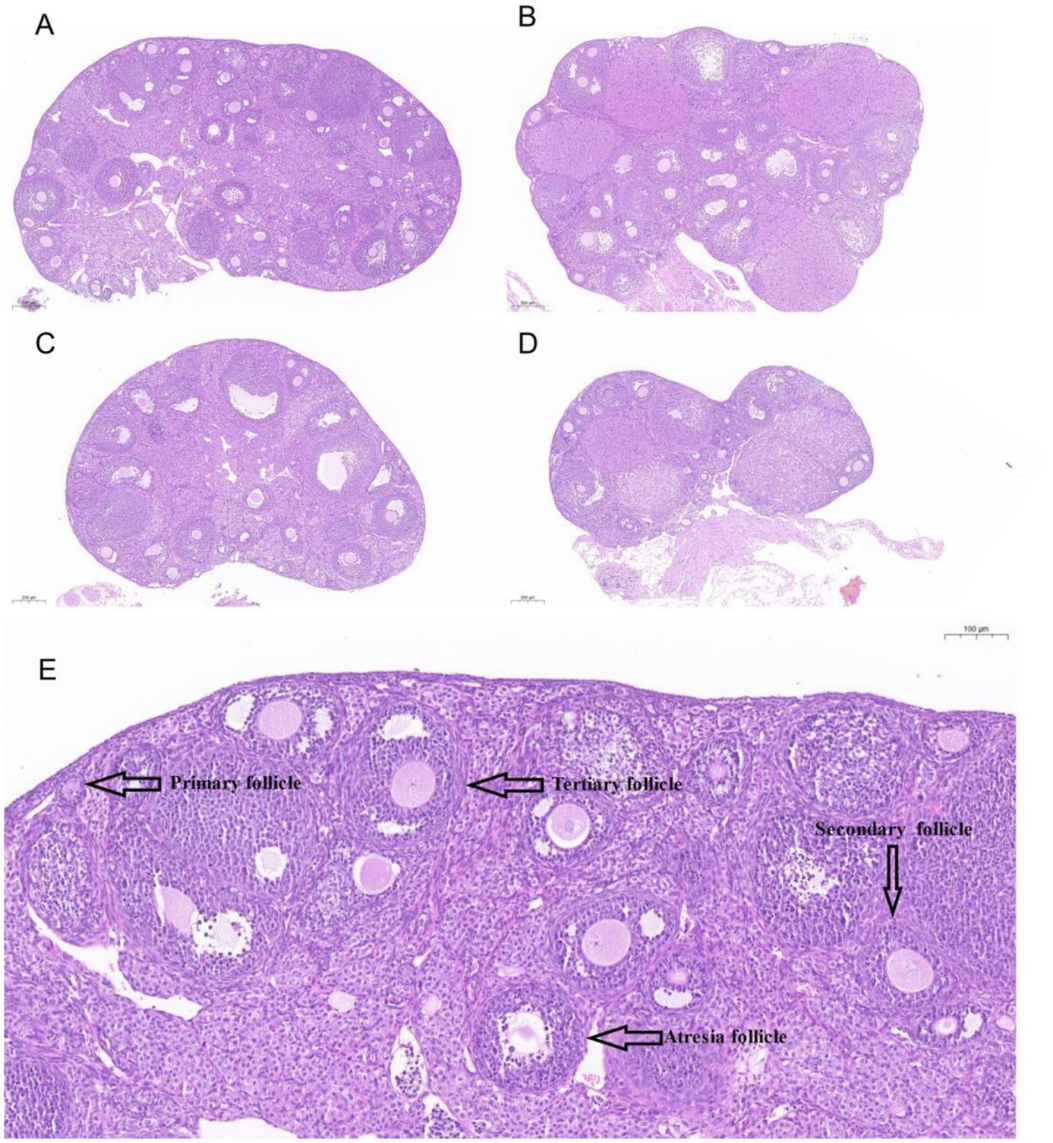
The ovary sections of mice stained by HE (Hematoxylin-Eosin). **(A–D)** indicated mice were treated with 0%, 0.75%, 1.5%, and 3.0% H_2_O_2_. **(E)**. The labels of different follicles at morphology and classification.

**Figure 4:**
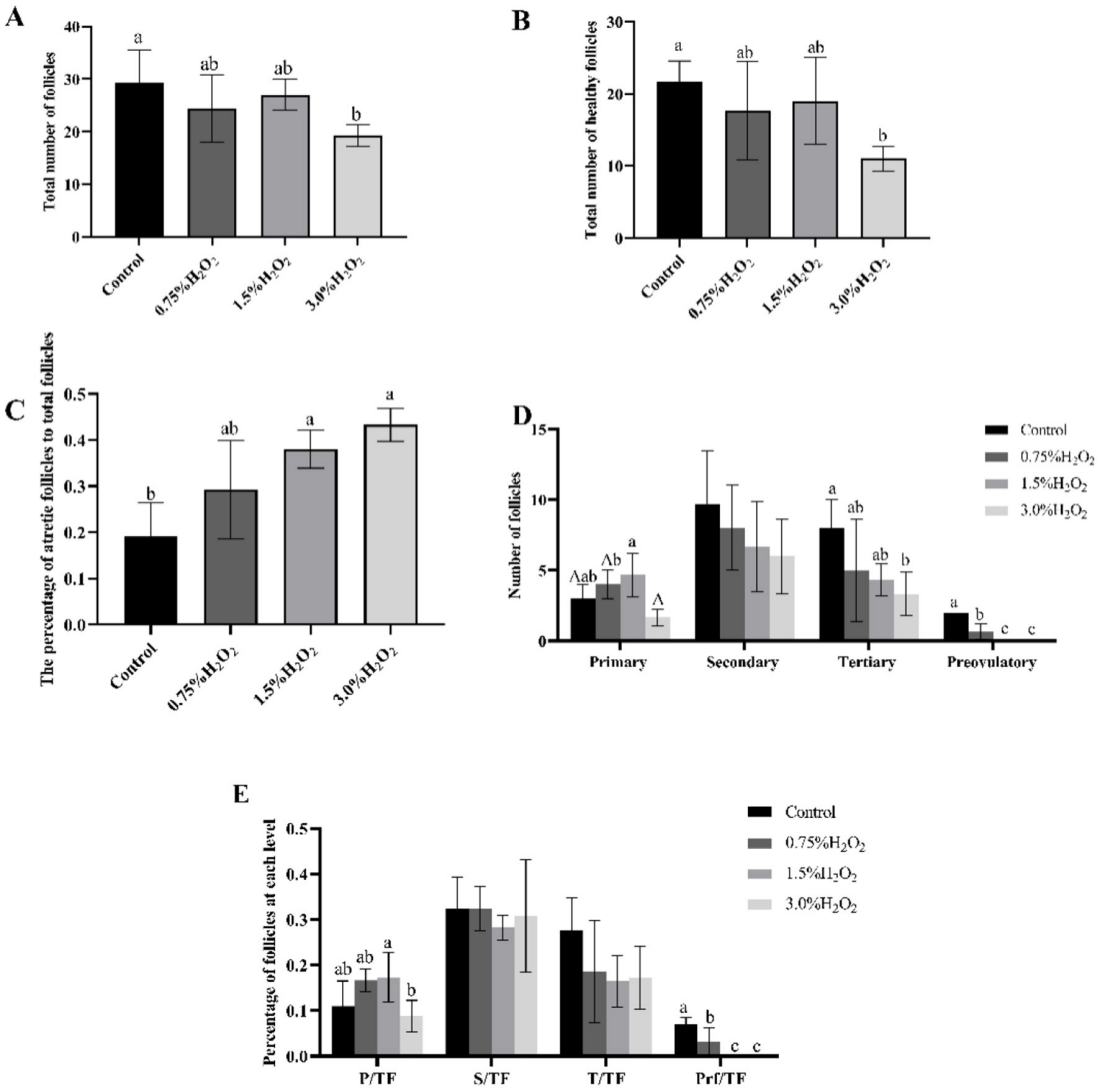
**(A)** The total number of follicles in four different H_2_O_2_-treated groups with control, 0.75%, 1.50%, and 3.0% H_2_O_2_. **(B)** The number of healthy follicles in four treated groups. **(C)** The ratio of atretic follicles to total number of follicles in four treated with control, 0.75%, 1.50%, and 3.0% H_2_O_2_. **(D)** The number of follicles in each category in four treated groups. **(E)** The proportion of each level follicle to total follicles in four treated groups. P, Primary follicles; S, Secondary follicles; T, Tertiary follicles; Prf, Preovulatory follicles; TF, The total number of follicles. Letters indicate statistically significant differences, a and b indicate *P* < 0.05, and A indicate *P* < 0.01.

To further assess the impact of H_2_O_2_-induced damage on the ovary, the ovarian morphology of female mice from various treatment groups was examined. Figures 3A–D provides a graphical representation of ovarian morphology and follicular development across different follicle stages. Additionally, Figure 3E distinctly illustrates the morphology and classification of the various follicle types. Morphological analysis of the ovarian sections, under identical magnification, revealed substantial damage in the ovaries of mice from group C compared to the control group. In conjunction with Figures 4A, B, the data indicate a marked decrease in both the total number of follicles and the number of healthy follicles in the treated groups compared to the control. Notably, group D exhibited the most severe ovarian damage, with significant oxidative damage also observed in the ovaries of group C, relative to the control group. As illustrated in Figure 4C, increasing H_2_O_2_ concentrations correlated with a rise in the proportion of ovarian atretic follicles. The control group's ovaries had significantly fewer percentages of atretic follicles compared to those in experimental group C (*P* < 0.05). To assess the developmental status of ovarian follicles and their potential reproductive capacity, the study analyzed the number of follicles in each category. Figure 4D revealed that the number of primary follicles in group C was significantly higher than in groups B (*P* < 0.05) and D (*P* < 0.01). The number of secondary and tertiary follicles exhibited a decreasing trend with rising H_2_O_2_ concentrations. Figure 4E demonstrates that the proportion of primary follicles relative to the total follicle count was significantly higher in group C compared to group D (*P* < 0.05). Additionally, no preovulatory follicles were observed in either the H_2_O_2_-treated group C or D (Figure 4D). In contrast, the untreated control group exhibited the highest number and percentage of preovulatory follicles across all groups (Figures 4C, E) (*P* < 0.05). These findings indicate that as the concentration of H_2_O_2_ increased, both the total number of follicles and the number of pre-ovulatory follicles decreased progressively. This suggests that elevated levels of oxidative stress may impair follicular development and negatively impact ovulation.

## 5 Discussion

In this study, mice were injected with varying concentrations of H_2_O_2_ intraperitoneally to establish an ovarian oxidative stress model. The validity of this model was confirmed through multiple measures, including analyzing serum antioxidant enzyme levels, lipid oxidation markers, and histological analysis of ovarian tissue sections. In order to ensure consistency among the samples, we selected mice of comparable age, sex, weight, and growth for the experiment. Given the variability in sensitivities and specificities of different tests for measuring oxidative stress and antioxidant activity, we carefully selected the most suitable approach based on the analysis of the mice blood samples and the H_2_O_2_ treatment. This meticulous selection aims to enhance the accuracy of the results. For the detection of MDA production in mice serum resulting from H_2_O_2_ treatment, we employed the TBA assay. MDA content, a critical indicator of oxidative stress, reflects the rate and intensity of lipid peroxidation and indirectly indicates tissue damage. The results of our study showed that serum MDA levels were significantly higher in group C mice compared to the control group, confirming that H_2_O_2_ induces a stress response. Interestingly, as the H_2_O_2_ concentration increased, MDA levels increased, suggesting a correlation with the elevated antioxidant enzyme systems in the body.

Additionally, serum SOD activity was measured to further validate the reliability of the oxidative stress model. SOD activity in mice was assessed using the WST-1 technique, which measures the enzyme's ability to inhibit nitrogen oxide-catalyzed ferrous cyanide oxidation at low concentrations, thereby reducing the rate of oxidation (25). Higher SOD expression was observed in the treated mice, although a decreasing trend was noted in the group exposed to higher H_2_O_2_ concentrations, indicating a threshold for cellular damage (26). GSH-Px works in conjunction with SOD to regulate H_2_O_2_ levels in the body (26). Along with CAT, GSH-Px inhibits the formation of highly toxic hydroxyl radicals, thereby mitigating the effects of H_2_O_2_ (27). GSH-Px also reduces hydroperoxides derived from polyunsaturated fatty acids, counteracting the harmful effects of lipid peroxidation. Following H_2_O_2_ injections at varying concentrations, intra-serum GSH-Px activity decreased, with significant reductions observed in group C compared to the control, confirming oxidative stress *in vivo*. This reduction in GSH-Px may also explain the observed decline in MDA levels. CAT plays a crucial role in maintaining cellular homeostasis due to its kinetic and thermodynamic properties, ensuring efficient catalytic turnover (28). CAT activity is modulated by H_2_O_2_ concentrations, catalyzing its disproportionation at high levels and triggering peroxidation pathways at low H_2_O_2_ concentrations (26, 28, 29). This allows CAT to oxidize hydrogen donors and reduce H_2_O_2′_s harmful effects. In this study, CAT levels in the serum of treated mice showed an initial increase followed by a decrease compared to the control group, reflecting disruption of the organism's antioxidant defense system. Although no significant changes in CAT expression were detected, the trend suggests oxidative stress. Moreover, stress-induced mortality was significantly higher in mice receiving 3% H_2_O_2_ intraperitoneal injections, further confirming the oxidative damage caused by H_2_O_2_ exposure. These results reflected the importance of controlling H_2_O_2_ concentrations in building the model to avoid excessive oxidative stress and associated damages.

To further confirm the effects of oxidative stress on ovarian tissues, histological staining was performed. The oocytes inside the follicle go through a process of cell division and maturation to become a mature egg during the stage of follicular development (24). The ovarian morphology and follicle content provided insight into the degree of stress. In studies of ovarian function, its follicle count can be an important indicator (30). Oxidative stress can contribute to a decrease in follicle number, which may be due to free radicals or lipid peroxidation causing cell damage and eventually apoptosis (31). Besides, oxidative stress is an important cause of follicular atresia (32). In the current investigation, mice administered a 1.5% H_2_O_2_ intraperitoneally. While the overall number of follicles in the ovaries was not significantly lower than in the control group, the percentage of atretic follicles was significantly higher. The life activity of the organism is a complex regulatory network. Numerous biological mechanisms are linked to follicular atresia. Apoptosis in the ovary regulates the process of follicular atresia and affects follicular degeneration, folliculogenesis and oogenesis (33). It has been shown that oxidative stress can cause apoptosis of granulosa cells and oocytes through the mitochondrial pathway, endoplasmic reticulum stress pathway, and death receptor pathway, and both apoptosis and autophagy are important mechanisms of oocyte and granulosa cell death (34). As a result, as the data in Figure 4C demonstrate, the proportion of atretic follicles is also a significant predictor of ovarian oxidative stress. Taken together, the results suggest that the injections in Group C mice (injected at a concentration of 1.5%) are more suitable for use in the study of the mechanisms underlying the onset of oxidative stress in the ovary.

The purpose of this study was to evaluate the effects of various treatment doses on mice ovarian oxidative stress. Compared with other animal models, mice models have the characteristics of low cost, easy operation, small size, rapid reproduction, easy breeding and physiopathology close to human (35). In addition, we found that although mice models have been applied in otitis media, degenerative kyphoscoliosis, renal injury and breast cancer, there is a lack of relevant studies in ovarian (36–39). H_2_O_2_, with its dual oxidative and reductive properties, acts differently depending on the environment and is a known indicator of oxidative stress (27). This study demonstrated the ability of H_2_O_2_ to induce oxidative stress in mice ovaries, providing a valuable platform for future research on female reproductive diseases caused by oxidative stress.

In our study, although the primary focus was on the ovarian effects, systemic measurements of oxidative stress markers were conducted, reflecting a broader, non-specific oxidative stress response. It is crucial to recognize that H_2_O_2_ has the potential to induce cytotoxic effects across multiple organ systems. Therefore, we recommend that future research should investigate the systemic impact of H_2_O_2_ to achieve a more comprehensive understanding of its effects on various organs. The current study has some limitations that should be taken into account in future research. Firstly, our study lacks detailed metrics for growth parameters, such as body length, organ weight ratios, and developmental milestones. These parameters are important for assessing the overall growth and development of the subjects. Secondly, reproductive outcomes, including fertility and litter size in female mice post-treatment, were not monitored. These outcomes are crucial for understanding the long-term reproductive impacts of oxidative stress and should be included in future studies. Additionally, the study did not provide measurements of ovary weight or the ratio of ovary weight to body weight. These measurements are essential for establishing an animal model of ovarian oxidative stress induced by H_2_O_2_. Therefore, future studies must incorporate these parameters to provide a more comprehensive understanding of the effects of oxidative stress on ovarian function.

## 6 Conclusion

Based on our findings, we have determined that injecting group C mice with a 1.5% concentration of H_2_O_2_ is the most appropriate approach for studying the mechanism of oxidative stress in the ovary. High quantity of H_2_O_2_ can cause irreparable harm to an organism, which poses challenges for further research. It is evident that establishing a model of oxidative stress caused by H_2_O_2_ in mice ovaries is feasible. Developing a specific model will provide researchers with a stronger foundation for understanding oxidative stress induced by H_2_O_2_ in ovaries facilitating the treatment strategies against reproductive illness.

## Data Availability

The original contributions presented in the study are included in the article/supplementary material, further inquiries can be directed to the corresponding authors.

## References

[1] Aitken RJ. Impact of oxidative stress on male and female germ cells: implications for fertility. Reproduction. (2020) 159:R189–201. 10.1530/REP-19-0452 31846434

[2] Agarwal A Gupta S Sekhon L Shah R. Redox considerations in female reproductive function and assisted reproduction: from molecular mechanisms to health implications. Antioxid Redox Signal. (2008) 10:1375–403. 10.1089/ars.2007.1964 18402550

[3] Al-Gubory KH Fowler PA Garrel C. The roles of cellular reactive oxygen species, oxidative stress and antioxidants in pregnancy outcomes. Int J Biochem Cell Biol. (2010) 42:1634–50. 10.1016/j.biocel.2010.06.001 20601089

[4] Zhang B Qu G Nan Y Zhou EM. Ovarian oxidative stress induced follicle depletion after zona pellucida 3 vaccination is associated with subfertility in BALB/c mice. Front Vet Sci. (2022) 9:814827. 10.3389/fvets.2022.814827 35252419 PMC8894603

[5] Hajam YA Rani R Ganie SY Sheikh TA Javaid D Qadri SS. Oxidative stress in human pathology and aging: molecular mechanisms and perspectives. Cells. (2022) 11:552. 10.3390/cells11030552 35159361 PMC8833991

[6] Cai M Li Q Cao Y Huang Y Yao H Zhao C. Quercetin activates autophagy to protect rats ovarian granulosa cells from H_2_O_2_-induced aging and injury. Eur J Pharmacol. (2024) 966:176339. 10.1016/j.ejphar.2024.176339 38272342

[7] Lee JC Son YO Choi KC Jang YS. H2O2 induces apoptosis of BJAB cells due to formation of hydroxyl radicals via intracellular iron-mediated Fenton chemistry in glucose oxidase-mediated oxidative stress. Mol Cells. (2006) 22:21–9. 10.1016/S1016-8478(23)17386-9 16951546

[8] Wu H Ye N Huang Z Lei K Shi F Wei Q. Dietary curcumin supplementation relieves hydrogen peroxide-induced testicular injury by antioxidant and anti-apoptotic effects in roosters. Theriogenology. (2023) 197:46–56. 10.1016/j.theriogenology.2022.10.038 36470109

[9] Sugino N. Reactive oxygen species in ovarian physiology. Reprod Med Biol. (2005) 4:31–44. 10.1007/BF03016135 29699208 PMC5904601

[10] Yan F Zhao Q Li Y Zheng Z Kong X Shu C. The role of oxidative stress in ovarian aging: a review. J Ovarian Res. (2022) 15:100. 10.1186/s13048-022-01032-x 36050696 PMC9434839

[11] Yi-Wen H Li-Na S Li-E H Yang Z Guo-Hong C Qi X. Vitamin E relieves the apoptosis of goose (Anser cygnoides) granulosa cells by inhibiting oxidative stress. J Agric Biotechnol. (2023) 31:1659–70. 10.3969/j.issn.1674-7968.2023.08.010

[12] Xiufang L Zhongqing W Lei Z Xiaoping Z Haiyan X Fang L. Kidney-tonifying Chinese medicine in alleviating oxidative stress in oocyte in older infertile women: a review. World J Tradit Chin Med. (2022) 17:3263–9. 10.3969/j.issn.1673-7202.2022.22.024

[13] Deng D Yan J Wu Y Wu K Li W. Morroniside suppresses H2O2-stimulated autophagy and apoptosis in rat ovarian granulosa cells through the PI3K/AKT/mTOR pathway. Hum Exp Toxicol. (2021) 40:577–86. 10.1177/0960327120960768 32954801

[14] Yang K Liu L Zhou H Xiao X Liu H. Advances on regulation of premature ovarian failure by oxidative stress and autophagy apoptosis. J Hunan Univ Chinese Med. (2021) 41:809–14. 10.3969/j.issn.1674-070X.2021.05.031

[15] Huang B Qian C Ding C Meng Q Zou Q Li H. Fetal liver mesenchymal stem cells restore ovarian function in premature ovarian insufficiency by targeting MT1. Stem Cell Res Ther. (2019) 10:362. 10.1186/s13287-019-1490-8 31783916 PMC6884777

[16] Lu J Wang Z Cao J Chen Y Dong Y. A novel and compact review on the role of oxidative stress in female reproduction. Reprod Biol Endocrinol. (2018) 16:80. 10.1186/s12958-018-0391-5 30126412 PMC6102891

[17] Khan A Dou J Wang Y Jiang X Khan MZ Luo H. Evaluation of heat stress effects on cellular and transcriptional adaptation of bovine granulosa cells. J Anim Sci Biotechnol. (2020) 11:1–20. 10.1186/s40104-019-0408-8 32095238 PMC7027041

[18] Khan A Khan MZ Dou J Umer S Xu H Sammad A. RNAi-mediated silencing of catalase gene promotes apoptosis and impairs proliferation of bovine granulosa cells under heat stress. Animals. (2020) 10:1060. 10.3390/ani10061060 32575551 PMC7341290

[19] Khan A Khan MZ Dou J Xu H Liu L Zhu H. SOD1 gene silencing promotes apoptosis and suppresses proliferation of heat-stressed bovine granulosa cells via induction of oxidative stress. Vet Sci. (2021) 8:326. 10.3390/vetsci8120326 34941853 PMC8708094

[20] Zha Z Liu SS Liu YJ Li C Wang L. Potential utility of natural products against oxidative stress in animal models of multiple sclerosis. Antioxidants. (2022) 11:1495. 10.3390/antiox11081495 36009214 PMC9404913

[21] Balmus IM Lefter R Ciobica A Cojocaru S Guenne S Timofte D. Preliminary biochemical description of brain oxidative stress status in irritable bowel syndrome contention-stress rat model. Medicina (Kaunas). (2019) 55:776. 10.3390/medicina55120776 31817740 PMC6956041

[22] Chen Z Xing T Li J Zhang L Jiang Y Gao F. Hydrogen peroxide-induced oxidative stress impairs redox status and damages aerobic metabolism of breast muscle in broilers. Poult Sci. (2021) 100:918–25. 10.1016/j.psj.2020.11.029 33518145 PMC7858176

[23] Zhong Y Ma T Fu Z Chen A Yu J Huang Y. Effects of hydrogen peroxide-induced oxidative stress on intestinal morphology, redox status, and related molecules in squabs. Animals. (2023) 13:749. 10.3390/ani13040749 36830536 PMC9952636

[24] Yisireyili M Alimujiang A Aili A Li YL Yisireyili S Abudureyimu K. Chronic restraint stress induces gastric mucosal inflammation with enhanced oxidative stress in a murine model. Psychol Res Behav Manag. (2020) 13:383–93. 10.2147/PRBM.S250945 32440237 PMC7210023

[25] Offer T Russo A Samuni A. The pro-oxidative activity of SOD and nitroxide SOD mimics. FASEB J. (2000) 14:1215–23. 10.1096/fasebj.14.9.1215 10834943

[26] Djordjević VV Kostić J Krivokapić Ž Krtinić D Ranković M Petković M. Decreased activity of erythrocyte catalase and glutathione peroxidase in patients with schizophrenia. Medicina. (2022) 58:1491. 10.3390/medicina58101491 36295651 PMC9609318

[27] Shen Y Shen Z Li P Chen Z Wei B Liu D. Protective activity of Malus doumeri leaf extract on H(2)O(2)-induced oxidative injury in H9C2 rat cardiomyocytes. Front Cardiovasc Med. (2022) 9:1005306. 10.3389/fcvm.2022.1005306 36187007 PMC9523085

[28] Sepasi Tehrani H Moosavi-Movahedi AA. Catalase and its mysteries. Prog Biophys Mol Biol. (2018) 140:5–12. 10.1016/j.pbiomolbio.2018.03.001 29530789

[29] Di Marzo N Chisci E Giovannoni R. The role of H2O2 in redox-dependent signaling: homeostatic and pathological responses in mammalian cells. Cells. (2018) 7:156. 10.3390/cells7100156 30287799 PMC6211135

[30] Mahmoodi M Soleimani Mehranjani M Shariatzadeh SM Eimani H Shahverdi A. N-acetylcysteine improves function and follicular survival in mice ovarian grafts through inhibition of oxidative stress. Reprod Biomed Online. (2015) 30:101–10. 10.1016/j.rbmo.2014.09.013 25458850

[31] Myers M Britt KL Wreford NG Ebling FJ Kerr JB. Methods for quantifying follicular numbers within the mouse ovary. Reproduction. (2004) 127:569–80. 10.1530/rep.1.00095 15129012

[32] Bao T Yao J Zhou S Ma Y Dong J Zhang C. Naringin prevents follicular atresia by inhibiting oxidative stress in the aging chicken. Poult Sci. (2022) 101:101891. 10.1016/j.psj.2022.101891 35561460 PMC9111992

[33] Vital Reyes VS Téllez Velasco S Hinojosa Cruz JC Reyes Fuentes A. Ovarian apoptosis. Ginecol Obstet Mex. (2001) 69:101–7. 11387878

[34] Zhang J Ren Q Chen J Lv L Wang J Shen M. Downregulation of miR-192 alleviates oxidative stress-induced porcine granulosa cell injury by directly targeting Acvr2a. Cells. (2022) 11:2362. 10.3390/cells11152362 35954205 PMC9368079

[35] Emini Veseli B Perrotta P De Meyer GR Roth L Van der Donckt C Martinet W. Animal models of atherosclerosis. Eur J Pharmacol. (2017) 816:3–13. 10.1016/j.ejphar.2017.05.010 28483459

[36] Dewan KK Taylor-Mulneix DL Campos LL Skarlupka AL Wagner SM Ryman VE. model of chronic, transmissible otitis media in mice. PLoS Pathog. (2019) 15:e1007696. 10.1371/journal.ppat.1007696 30970038 PMC6476515

[37] Hu Z Tang Z Kiram A Li J Xu H Xu Y. The establishment of a mouse model for degenerative kyphoscoliosis based on senescence-accelerated mouse prone 8. Oxid Med Cell Longev. (2022) 2022:7378403. 10.1155/2022/7378403 35910839 PMC9329026

[38] Yu K Zhang J Cao Z Ji Q Han Y Song M. Lycopene attenuates AFB(1)-induced renal injury with the activation of the Nrf2 antioxidant signaling pathway in mice. Food Funct. (2018) 9:6427–34. 10.1039/C8FO01301B 30462120

[39] Yang L Yong L Zhu X Feng Y Fu Y Kong D. Disease progression model of 4T1 metastatic breast cancer. J Pharmacokinet Pharmacodyn. (2020) 47:105–16. 10.1007/s10928-020-09673-5 31970615

